# Dyeing and Characterization of Cellulose Powder Developed from Waste Cotton

**DOI:** 10.3390/polym11121982

**Published:** 2019-12-02

**Authors:** Linli Gan, Heng Guo, Zhiheng Xiao, Zhiwei Jia, Han Yang, Dan Sheng, Heng Pan, Weilin Xu, Yunli Wang

**Affiliations:** 1 State Key Laboratory of New Textile Materials and Advanced Processing Technologies, Wuhan Textile University, Wuhan 430200, China; 2 Institute for Frontier Materials, Deakin University, Waurn Ponds, VIC 3216, Australia; 3 College of Chemistry and Chemical Engineering, Wuhan Textile University, Wuhan 430200, China

**Keywords:** recycling of waste cotton, cellulose powder, dyeing properties, specific surface area, thermal analysis, DMA

## Abstract

In this study, waste cotton fibers were environmentally reused. First, they were milled into fine powders with particle sizes of around 30 µm and dyed for use as pigments. Dyeing properties of the cellulose powder were explored by determining the dye uptake, K/S value, and bath ratio. Among the various samples, powders with owf (on weight of fabric) of 0% dye (pristine cellulose powder), and 10% and 50% dyed powders were selected; and these powders were characterized by several methods to compare the properties of dyed and undyed cellulose. The surface morphologies of the powders were observed with a scanning electron microscope (SEM). Combining the SEM images with the Brunauer–Emmet–Teller (BET) data, it was found that the smaller the particle size, the larger is the surface area. In addition, the X-ray photoelectron spectroscopy (XPS) results revealed that with increasing dye concentration, the intensity of the C peak reduced, while those of O and S increased. Moreover, the main components of the dyed and undyed cellulose powders were found to be almost the same from the Fourier-transform infrared spectroscopy (FTIR) results. Finally, the dynamic mechanical analysis (DMA) data revealed that the loss modulus was significantly larger than the storage modulus, demonstrating that the material mainly undergoes viscous deformation.

## 1. Introduction

Cotton is the most widely utilized natural fiber in the world, accounting for over 82% of natural fibers globally consumed [1]. In 2016 and 2017, the world’s cotton consumption reached approximately 110 and 115 million bales, respectively [2]. Because of the increase in demand for cotton products, the amount of cotton currently available is not sufficient to meet the market demand. To conserve cotton fibers as a natural resource and to reduce energy and water consumption during production, it is highly significant to make full use of wasted scraps in the production process and reuse textile waste [3]. Notably, in spinning, 4%–8% of cotton fibers are lost during the opening and cleaning processes. Moreover, during combing and yarn production, the loss of cotton fibers is as high as 20%. If these waste fibers could be reused, it would create tremendous market potential for the textile industry [4].

Traditionally, recycling is achieved by drawing fabrics into yarns and fibers, and then, reassembling them into recyclable yarns for the production of textiles and other materials, such as nonwovens, carpet liners [5], sound insulation materials, thermal insulation materials [6], deodorizing materials, and filtering materials; thus, converting waste garments into recyclable ones [7].

Due to the special properties of large specific surface areas, superfine particle size, and excellent inertness [8], powders are widely used in energy, environmental protection, information, aviation, transportation, and many other fields. However, they are not commonly used in the textile industry. Because of the limited availability of textile resources and increasing amount of waste generated, it is highly significant to recycle waste textiles into new materials. Some researchers have generated powders from natural fibers and explored their further applications. For instance, Xu et al. [9] crushed wool fibers with diameters of 25 µm into a fine powder with a particle size of around 2 µm after treating them with a 0.5% NaClO solution. Huang et al. [10] and Xu et al. [11] blended down powder and wool powder with polypropylene to produce pellets and then hot-pressed them into films, respectively. Superfine down powder has been coated on polypropylene to produce dyeable fibers [12]. Kazemimostaghim et al. [13] decreased the particle sizes of silk powders from 7 μm to 200 nm by bead milling. Although some reports of transformation of natural fibers into powders are available, there are hardly any on the properties of dyed cotton powder. Most dye-based studies are focused on dyeing fabrics under different conditions. For example, Khatri et al. [14] studied the effect of a dye solution’s ionic strength on the dyeing of cotton fabric. Tang et al. [15] reported polyethylene glycol-based reverse micellar dyeing of cotton fabric. Cleaner coloration of cotton fabric with reactive dyes using a pad-batch-steam dyeing process was demonstrated by Shu et al. [16]. Some methods have been developed by researchers to solve specific problems [17], such as dyeing effluents [18], dye agglomeration [19], and poor dye uptake during dyeing of fabrics [20].

To the best of our knowledge, studies on the dyeing theory and dyeing properties of cellulose powder can hardly be found in the existing literature. Therefore, in this work, we studied the dyeing properties of cellulose powder; this study was expected to provide a new method to recycle large quantities of waste cellulose powders and a potential route to applying dyed cellulose powders as a new class of pigments for printing. Accordingly, the air, water, and soil pollution caused by the textile industry could effectively be controlled and waste textiles could be reused, thereby creating great market value.

## 2. Experimental

### 2.1. Materials

We used: *N′N*-dimethylformamide, acetone, sodium chloride, and anhydrous sodium carbonate (China Chemical Reagent Limited Company, Shanghai, China). Additionally, Reactive Red B-2BF (Tianjin Development Zone Letai Chemical Limited Company, Tianjin, China), cellulose powder (Wuhan Textile University, Wuhan, China), and cotton fabric (Hunan Huasheng Zhuzhou Xuesong Limited company, Hunan, China).

### 2.2. Milling Method of Cellulose Powder

Cellulose fiber was first cut into about 3 mm short fiber by a rotary blade, and then ground on a special grinding plate made in Wuhan Textile University. The plate has the characteristics of low heat production, good abrasion resistance, and the fiber can be ground superfine [9].

### 2.3. Effect of Dye Process on Dyeing Properties of Cellulose Powder

**Figure 1:**
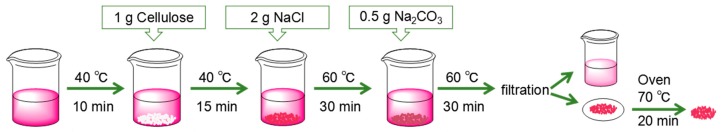
Diagram of dyeing process of cellulose powder using dyes of Reactive Red B-2BF.

The concentrations of NaCl and Na_2_CO_3_ used in the experiment shown in Figure 1 were 40 g/L and 10 g/L, respectively. To study the effect of dye dosage, the bath ratio was set as 1:50. To explore the effect of bath ratio on the dyeing properties, the dye dosage was set to 5% on weight of fabric (owf). To determine the salt effect, a bath ratio of 1:50 and an owf of 5% were used. Different parameters were set according to the variables. Dyeing solutions with different concentrations (1%, 10%, 30%, and 50% owf) were prepared to distinctly dye 1 g of cotton fabric by the same dyeing process. An XH-KG55B dyeing machine (Foshan huaboxing Technology Limited company, Guangdong, China) was employed for the entire dyeing process. The absorbance of the initial and residual solutions was measured using a V-5600 ultraviolet-visible spectrophotometer.

### 2.4. Characterization of Cellulose Powder Before and After Dyeing

The K/S values of the dyed powder and dye solution were determined using Datacolor110 (Shenzhen Xingke Instrument Limited company, Guangdong, China) with an illuminant D65 and CIE (Commission Internationale de L’Eclairage) 10° standard observer.

Scanning electron microscopy (SEM, HITACHI S-3000, Hitachi, Japan) was performed to observe the morphology of dyed cellulose powder after coating with gold at an acceleration voltage of 5 kV and magnification of 1000 times.

The specific surface area of cellulose powder was determined by the Brunauer–Emmett–Teller (BET) technique with TriStar 3000 (Micromeritics Instrument Company, Norcross, GA, USA). Typically, about 0.3 g of the specimen was placed in a glass analysis tube and degassed under nitrogen at 100 °C for 90 min to ensure that the surface was clean and the adsorbent was ready for analysis. The analysis tube was weighed and vacuum tested prior to BET nitrogen gas adsorption analysis. The sample was maintained at cryogenic conditions during the measurement.

Thermal gravity analysis (TGA) of the dyed powders was performed with a TG 209 instrument (NETZSCH, Selb, Germany) under a nitrogen atmosphere (gas flow of 40 mL/min) in the temperature range of 25–500 °C at a heating rate of 20 °C/min.

To examine the differences in the composition between the original and dyed powders, X-ray photoelectron spectroscopy (XPS) was performed on an ESCALAB 210 spectrometer (VG scientific, MA, USA); the C1s, O1s, and S2p spectra were recorded in the range of 0–1100 eV.

To further determine the chemical compositions of the samples, Fourier-transform infrared (FTIR) spectroscopy (Nicolet Magna-IR 560, Nicolet, Madison, WI, USA) was performed. ATR mode was used to test the samples. The spectral resolution was 4 cm^−1^ and the number of scans performed per sample was 128.

Dynamic mechanical analysis Q800 (DMA, TA instruments, DE, USA) was performed to explore the relationship between temperature and loss and storage moduli and determine the deformation properties in the temperature range of 25–250 °C under an N_2_ atmosphere at heating rate of 2 °C/min.

## 3. Results and Discussion

### 3.1. Influence of Bath Ratio and owf on Dyeing Properties of Cellulose Powder

**Figure 2:**
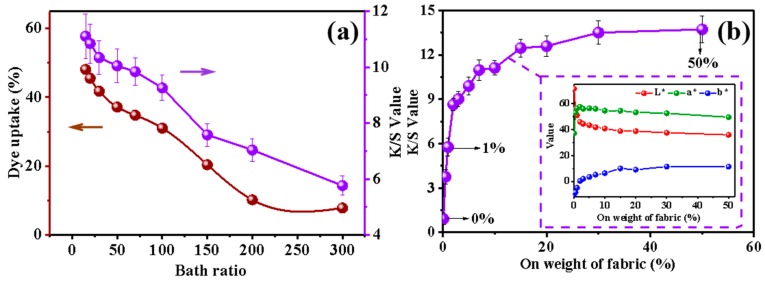
(**a**) Influence of bath ratio on K/S value (purple Y-axis pointed by the purple arrow) and dye uptake (brown Y-axis pointed by the brown arrow). (**b**) Relationship between on weight of fabric (owf) and K/S value and the L*, a*, and b* value.

The effect of bath ratio and on weight of fabric (owf) on dyeing properties are presented in Figure 2. At the same owf, the dye uptake and K/S value of cellulose powder distinctly decrease with increasing bath ratio (Figure 2a). The bath ratio directly determines the concentration of dyes and chemicals in the dyeing system. The smaller the bath ratio, the higher the concentration of dyes and auxiliaries in the dyeing solution, resulting in faster dyeing and a higher dye adsorption capacity.

It can be seen in Figure 2b that the K/S value rapidly increases first and gradually becomes constant as the owf increases, which indicates that the color of the dyed cellulose powder becomes deeper and eventually tends to be saturated. In addition, from the CIELab graph of L*, a*, and b* value, the three values of color change can be more clearly observed. As the owf increases, L* decreases, which means the color is darker, while a* and b* become larger, meaning that the color is redder and yellower. After 30% owf, the L*, a*, and b* all tend to stay the same, corresponding to the result of K/S.

Because the K/S value is the largest and most stable at 50% owf, and the K/S value at 1% owf is almost half of the largest K/S value, the 1% and 50% owf dyed cellulose powders were used for further tests and compared with the undyed cellulose powder.

### 3.2. The Influence of Salt on Dyeing Efficiency and the Effect of owf on Dye Adsorption

**Figure 3:**
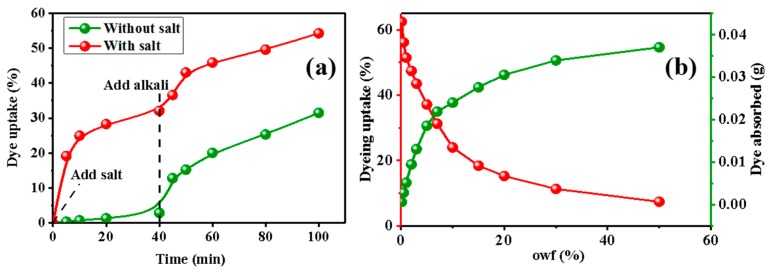
(**a**) Influence of salt on dye uptake. (**b**) Influence of owf on dye uptake (red Y-axis and red curve) and dye adsorbed (green Y-axis and green curve).

The two graphs in Figure 3 show the influence of salt on the dyeing efficiency and the effect of owf on dye adsorption. As shown in Figure 3a, salt influences the dye efficiency. Before the addition of salt into the dye solution, the dye uptake increases very slowly, while it rapidly increases after the addition of salt in the very first stage. The addition of salt as a neutral electrolyte not only weakens the Coulombic forces of the dye, but also increases the concentration of sodium ions in the dye solution, thereby reducing the concentration difference near the interface between the dye and the powder and increasing the diffusion rate of the dye. At the same time, the addition of electrolyte can weaken the solubility of the dye; thus, increasing the dye adsorption density and dye uptake. Nevertheless, cellulose powder increasingly adsorbs more dye with time and the dye uptake decreases as the concentration of the dye solution decreases. Subsequent to the addition of alkali at 40 min into both dye baths, the dye uptake increases further. This is attributable to an increase in the pH of the dye solution caused by the alkali agent. Anion concentration of cellulose rapidly increases, and subsequently, cellulose reacts with the reactive dye groups via nucleophilic substitution and nucleophilic addition reactions, promoting dye fixation. As it can be seen in the two graphs, the dye uptake is still low. It is a significant action—further improving the dying uptake exhaustion so as to decrease dye pollution.

**Figure 4:**
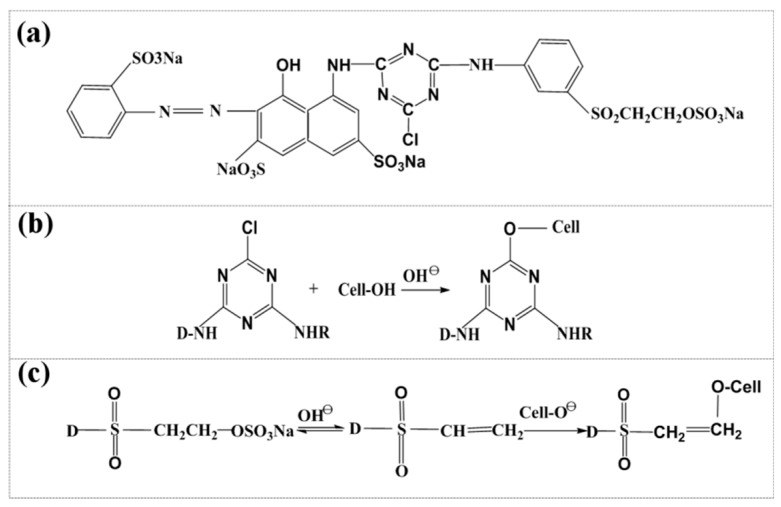
(**a**) Chemical structure of B-2BF. (**b**) Nucleophilic substitution reaction between B-type reactive dye and cellulose. (**c**) Nucleophilic addition reaction between B-type reactive dye and cellulose.

Reactive Red B-2BF, a B-type reactive dye, was used in the experiment. The molecular structure of the dye [21] is shown in Figure 4a. The nucleophilic substitution and nucleophilic addition reactions occurring between the B-type reactive dye and cellulose are presented in Figure 4b,c. Figure 1d is the sketch map of dye’s micromechanism.

B-type reactive dye is a kind of mixed reactive dye with high reactivity. The connecting group of the B-type reactive dye is 1-chlorotriazine. Vinyl sulfone sulfate was introduced into the dye matrix to provide it with the advantages of both active groups [22]. In addition, due to the strong electron-attracting effect of sulfonyl groups in the same conjugate system [23], the reactivity of the monochlorotriazine group in this composite structure was higher than that in a single structure [24]. These dyes exhibit the following advantages: high solubility, stable dyeing performances, outstanding levelness and permeability, excellent dyeing repeatability and compatibility, and strong adaptability to changes in the dyeing process. Cellulose fibers can be dyed at 60–80 °C. Under the action of alkaline substances, the mixed bi-functional groups of cellulose fibers can form covalent bonds with the hydroxyl groups in cellulose molecules to fix dyes [25].

Because of the bi-functional group structure of the B-type reactive dye, both nucleophilic substitution and nucleophilic addition reactions occur during the dyeing process of cellulose powder, attributable to dyeing and fixation via covalent bonds. Because the electronegativity of nitrogen atoms on the aromatic heterocycles of active groups is stronger than that of carbon atoms, the electron cloud density of each carbon atom on the heterocycles is lower, resulting in a partial positive charge [26]. The high electronegativity of chlorine atoms connected to the carbon atoms induces an inductive effect, leading to stronger positive charges on the carbon atoms. Thus, carbon atoms on aromatic heterocycles are more vulnerable to attack by nucleophilic reagents and nucleophilic substitution reactions occur [27]. The nucleophilic substitution and nucleophilic addition reactions between the B-type reactive dye and cellulose are illustrated in Figure 4b,c.

As presented in Figure 3b, with increasing owf, the amount of absolute dye adsorbed by cellulose powder increases, while the dye uptake reduces. When the dye concentration reaches a certain level, the aggregation of dye molecules in the dyeing solution rapidly increases, which increases the energy required for depolymerization of the dye, making dyeing more difficult. Meanwhile, as the amount of dye adsorbed becomes saturated, the dye uptake gradually decreases because cellulose powder adsorbs less dye molecules.

### 3.3. Dye Adsorption of Cotton Powder and Cotton Fabric with Different owf

**Figure 5:**
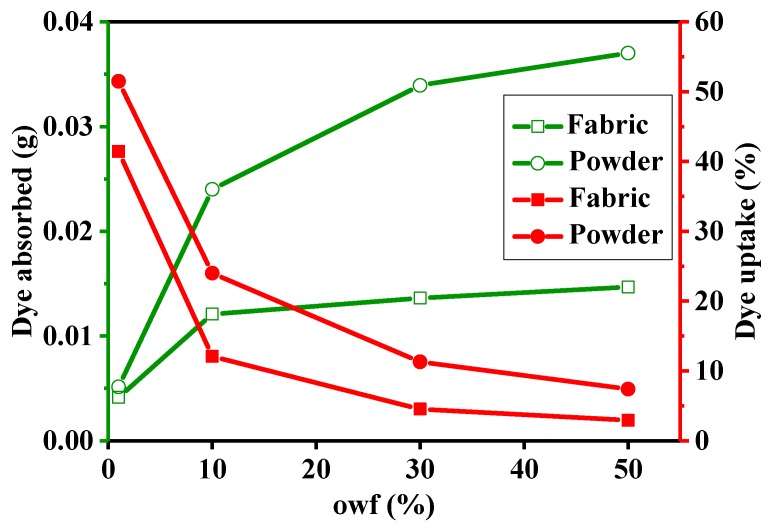
Dye adsorption of powder and fabric with different owfs.

The dyeing performances of cellulose powder and cotton fabric in different dye solutions are compared in Figure 5. Although the physical state of cellulose fibers changes, cellulose powder retains its chemical structure. Therefore, cellulose powder and cotton cloth share the same dyeing mechanism. As shown in Figure 5, even if cotton powder and cotton fabric share the same owf and weight, the powder achieves a higher dye uptake and adsorbs a larger amount of dye. Compared to a piece of cotton fabric, cotton powder has a larger specific surface area; therefore, the adsorption performance of cellulose powder is much higher than that of cotton fabric. During dyeing, the powder sufficiently swells and the dye rapidly diffuses into the powder. Hence, the dye adsorption capacity of cotton powder is larger than that of cotton fabric. In addition, with increasing owf, the variations in dye uptake and dye adsorption for cellulose fabric and powders are consistent with the result presented in Figure 3b.

### 3.4. Powder Morphology and Particle Size

**Figure 6:**
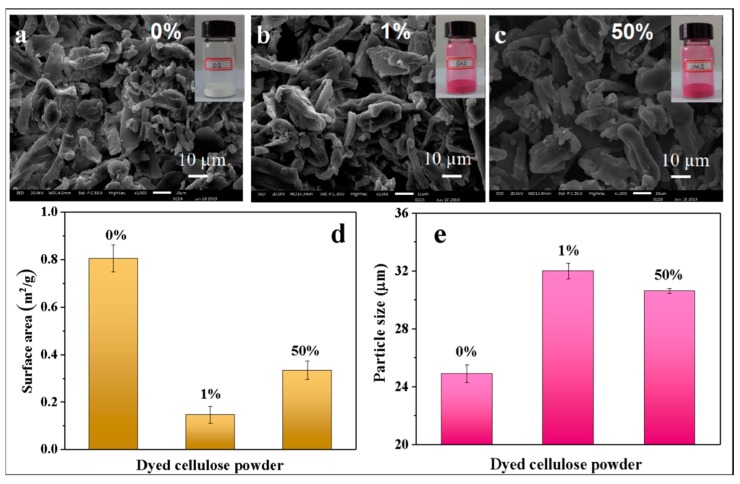
(**a–c**) SEM images of cellulose powders with varied dye uptake. (**d**) Particle size of cellulose powders with varied dye uptake. (**e**) Surface areas of cellulose powders with varied dye uptake.

The mean particle sizes of powders were statistically calculated after manually determining the sizes from the SEM images. The SEM images of the original cellulose powder and dyed powder are presented in Figure 6a–c. Due to the physical grinding process performed on a millstone, the powders were subjected to friction and shearing force randomly. It can be observed in the three images that the powder surfaces are rough and irregular. Most powder particles are bar shaped and the particle size is about 30 µm. Obviously, the surface of the powder with 50% dye uptake is smoother than those with 0% and 1% dye uptake. At 50% dye concentration, dyes are more dispersed and uniformly adsorbed on cellulose powder. The more uniform the distribution of dyes on fibers, the higher the bonding probability between the dyes and fibers, and the better the fixation. Because of swelling of the polymer, the volume of the powder with higher dye uptake is larger. As can be seen from the electron microscopy images, the average particle sizes of 0%, 1%, and 50% dyed powders are about 25, 34, and 32 µm, respectively, as shown in Figure 6e. The specific surface areas of the three powder samples were determined by the BET technique, as shown in Figure 6d. As the powder particle size increases, the specific surface area decreases.

### 3.5. Composition Analysis of Dyed and Undyed Cellulose Powders

**Figure 7:**
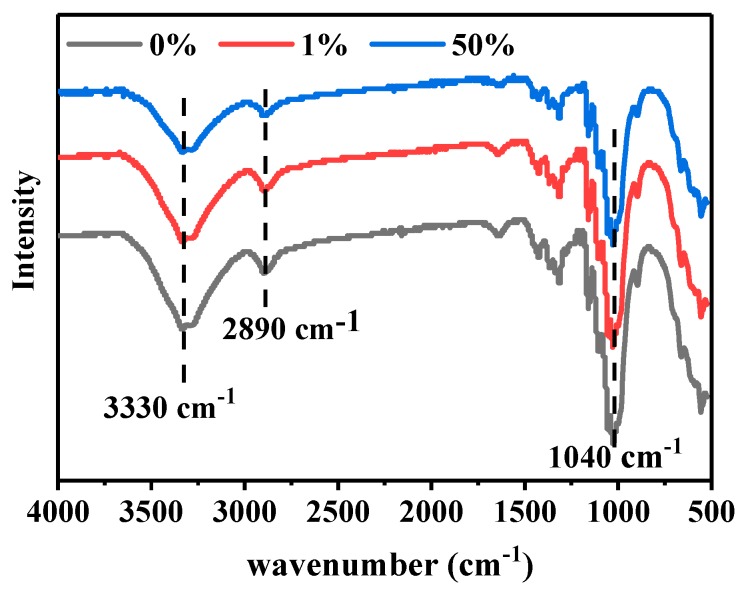
FTIR spectra of 0%, 1%, and 50% owf dyed cellulose powders.

FTIR analysis was carried out to verify the difference in the chemical composition between the dyed and undyed powders and the results are presented in Figure 7. As observed, the three spectra are similar and they exhibit similar peaks at 1040, 2890, and 3330 cm^−1^, corresponding to C–O, C–H, and O–H respectively. The peak due to the C–O group containing C and O, which are the main compositional elements of cellulose, is the strongest. The dyed cellulose powders do not exhibit any new peaks, because the amount of dye used in the dyeing process was very small.

**Figure 8:**
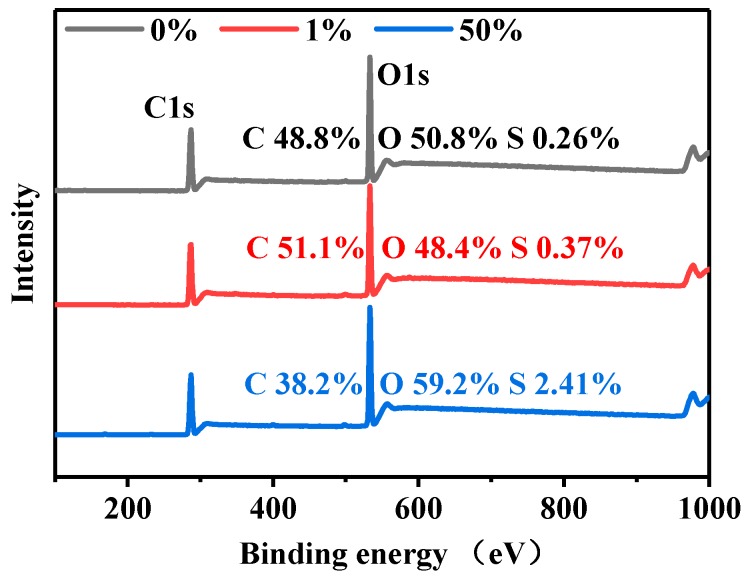
XPS profiles of 0%, 1%, and 50% owf dyed cellulose powders.

Further compositional analysis of the dyed cellulose powders was carried out by XPS (Figure 8). C1s and O1s peaks are observed at 286 and 529.6 eV respectively. The content of each element is consistent with the molecular weight calculated according to the formula. As the concentration of the dye increases, the intensity of the C peak reduces, while those of the O and S peaks increase. There is no apparent relationship between the dyed and undyed cellulose powders. This can be explained on the basis of the nucleophilic substitution and nucleophilic addition reactions between the B-type reactive dye and cellulose, as shown in Figure 4. It can also be inferred that in the dyeing process, mainly, nucleophilic substitution reaction occurs, because the nucleophilic addition reaction causes significant changes in the amounts of C and O, compared to that of S. Moreover, the peak for sulfur should be at 173 eV and the content for it changed a little. However, there is no obviously strong peak for sulfur in Figure 8, which should be attributed to the fact that the dye dosage adsorbed was relatively smaller than the amount of cellulose powder.

### 3.6. Thermal Gravity Analysis

**Figure 9:**
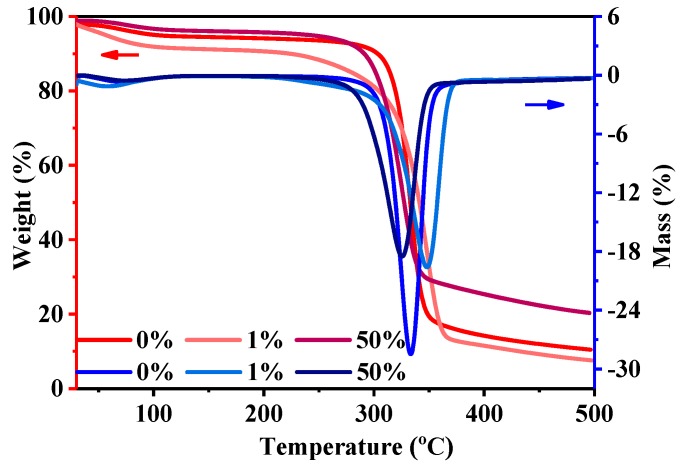
Thermal gravity graphs (Weight: red Y-axis pointed by the red arrow; Mass: blue Y-axis pointed by the blue arrow) of cellulose powders with owfs.

The TGA graphs of 0%, 1%, and 50% owf dyed cellulose powders are shown in Figure 9. The weight loss values for the samples are 76.2%, 77.2%, and 66.5%. The total weight loss of 50% dyed cellulose powder is less than those of the 0% and 1% owf dyed powders, indicating that the 50% owf dyed cellulose powder experienced the least thermal decomposition. It can be inferred that when dyes tend to saturate, they may protect partial cellulose powder from being decomposed. The derivatives of the TG graphs were plotted to clearly understand the thermal stability during heating. From the differential thermogravimetry curves of dyed cellulose powders, the thermal decomposition temperatures of 0%, 1%, and 50% owf dyed powders were determined to be 334, 348, and 324 °C, respectively. Fifty percent owf dyed cellulose powder decomposed at a lower temperature compared with 1% dyed powders; we speculate that the thermal decomposition temperatures of dyed cellulose powder become lower with higher dye dosage adsorbed.

### 3.7. DMA Measurements

**Figure 10:**
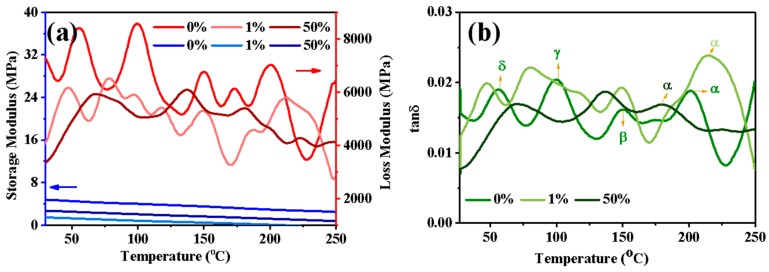
(**a**) Storage (blue Y-axis pointed by blue arrow and blue curves) and loss moduli (red Y-axis pointed by red arrow and red curves) and (**b**) tan δ plots of 0%, 1%, and 50% owf dyed cellulose powders and the α, β, γ, and δ transition.

It is obvious from Figure 10a that as temperature increases, the storage moduli of dyed and undyed cellulose powder decrease. The energy generated from elastic deformation decreases, which indicates that the resilience of cellulose powder decreases. In addition, the 0% owf dyed cellulose powder exhibits the highest storage modulus, indicating that the deformation capacity of cellulose powder decreases after dyeing. In addition, the undyed sample exhibits the largest loss modulus, indicating that the energy loss due to viscous deformation for 0% owf dyed cellulose powder is the largest and the viscosity of the sample is the highest of those of 1% and 50% owf dyed powders. As the loss modulus is much larger than the storage modulus, the material mainly undergoes viscous deformation, revealing that the material is a liquid [28]. This further verifies that powder has the property of a fluid, which has no specific shape and can flow and fly. It can be considered that powder is the transition state of a material between the fluid and solid states, although this is not clearly defined in physics.

Figure 10b presents the relationship between tan δ and temperature. tan δ can be determined using the following Equation [29],

$$\tan\delta =\frac{E^{\prime \prime }}{E^{\prime }}$$

where *E*″ is the loss modulus, *E*′ is storage modulus, δ is the loss angle, and tan δ is the loss tangent [30].

The tan δ curve shows a series of peaks, each corresponding to a specific relaxation process. The main transition is the α transition, and β, γ, and δ transitions are secondary transitions, which correspond to the movement of mobile units smaller than the chain segments, such as side groups, end groups, and short chain links, indicating a certain transition in the movement of polymer molecules occurs in these temperature regions. αtransition is the most important transition, indicating that the storage modulus is obviously reduced. At the same time, the molecular chain segment overcomes the environmental viscous motion and consumes energy, giving rise to peaks in the E″ and tan δ curves. As observed in Figure 10 b, the order of thermal decomposition temperature of the three cellulose powder is 1% > 0% > 50%, which is consistent with the TGA result (Figure 9).

## 4. Conclusions

The absolute dye exhaustion increases while the dye uptake decreases with increasing dye concentration. The bath ratio was found to be inversely proportional to the dye uptake up to a certain level. In addition, both salt and alkali positively influence the dye efficiency. The dye uptake of cellulose powder is obviously higher than that of cotton fabric. Notably, the 50% owf dyed cellulose powder shows a relatively smooth surface. Combining the BET results with the SEM images, it was confirmed that the smaller the particle size, the larger is the specific surface area. The XPS results show that with increasing dye concentration, the intensity of the C peak reduces, while those of O and S increase. In addition, the FTIR results reveal that the main components of dyed and undyed cellulose powders are the same. The DMA data show that the loss modulus is significantly larger than the storage modulus, demonstrating that the material mainly undergoes viscous deformation. The dyed cellulose powders can be used as pigments for printing and additives in polyurethane, etc., thereby facilitating environmental protection and conservation of the limited natural resources.

## References

[1] Baydar G., Ciliz N., Mammadov A. (2015). Life cycle assessment of cotton textile products in Turkey. Resour. Conserv. Recycl..

[2] Liu W., Liu S., Liu T., Liu T., Zhang J., Liu H. (2019). Eco-friendly post-consumer cotton waste recycling for regenerated cellulose fibers. Carbohydr. Polym..

[3] Yousef S., Tatariants M., Tichonovas M., Sarwar Z., Jonuškienė I., Kliucininkas L. (2019). A new strategy for using textile waste as a sustainable source of recovered cotton. Resour. Conserv. Recycl..

[4] Yuen C.W.M., Cheng Y.F., Li Y., Hu J.Y. (2009). Preparation and characterisation of nano-scale cotton powder. J. Text. Inst..

[5] Haule L.V., Carr C.M., Rigout M. (2016). Preparation and physical properties of regenerated cellulose fibres from cotton waste garments. J. Clean. Prod..

[6] Haule L.V., Carr C.M., Rigout M. (2014). Investigation into the removal of an easy-care crosslinking agent from cotton and the subsequent regeneration of lyocell-type fibres. Cellulose.

[7] Peña-Pichardo P., Martínez-Barrera G., Martínez-López M., Ureña-Núñez F., dos Reis J.M.L. (2018). Recovery of cotton fibers from waste Blue-Jeans and its use in polyester concrete. Constr. Build. Mater..

[8] Guan Y., Li Y. (2008). Fabrication of cotton nano-powder and its textile application. Chin. Sci. Bull..

[9] Xu W., Cui W., Li W., Guo W. (2004). Development and characterizations of super-fine wool powder. Powder Technol..

[10] Huang J., Liu X., Li W., Xu W. (2012). Preparation and characterization of polypropylene/superfine down powder blend films. J. Thermoplast. Compos. Mater..

[11] Xu W., Wang X., Li W., Peng X., Liu X., Wang X.G. (2007). Characterization of superfine wool powder/poly(propylene) blend film. Macromol. Mater. Eng..

[12] Liu X., Huang J., Huang J., Li W., Xu W. (2011). Coating superfine down powder on polypropylene for the production of dyeable fibers. Fibers Polym..

[13] Kazemimostaghim M., Rajkhowa R., Tsuzuki T., Wang X. (2013). Production of submicron silk particles by milling. Powder Technol..

[14] Khatri A., White M., Padhye R. (2018). Effect of Dye Solution Ionic Strength on Dyeing of Cotton with Reactive Dyes. Fibers Polym..

[15] Tang A.Y., Lee C.H., Wang Y.M., Kan C.W. (2019). A study of PEG-based reverse micellar dyeing of cotton fabric: Reactive dyes with different reactive groups. Cellulose.

[16] Shu D., Fang K., Liu X., Cai Y., Zhang X., Zhang J. (2019). Cleaner coloration of cotton fabric with reactive dyes using a pad-batch-steam dyeing process. J. Clean. Prod..

[17] Irfan M., Zhang H., Syed U., Hou A. (2018). Low liquor dyeing of cotton fabric with reactive dye by an eco-friendly technique. J. Clean. Prod..

[18] Xia L., Wang A., Zhang C., Liu Y., Guo H., Ding C., Wang Y., Xu W. (2018). Environmentally friendly dyeing of cotton in an ethanol-water mixture with excellent exhaustion. Green Chem..

[19] Lee C.H., Tang A.Y.L., Wang Y., Kan C.W. (2019). Effect of reverse micelle-encapsulated reactive dyes agglomeration in dyeing properties of cotton. Dyes Pigment..

[20] Acharya S., Abidi N., Rajbhandari R., Meulewaeter F. (2014). Chemical cationization of cotton fabric for improved dye uptake. Cellulose.

[21] Lewis D.M., Clark M. (2011). The chemistry of reactive dyes and their application processes. Handbook of Textile and Industrial Dyeing.

[22] Chakraborty J.N. (2014). Dyeing with reactive dye. Fundamentals and Practices in Colouration of Textiles.

[23] Burkinshaw S.M., Kabambe O. (2011). Attempts to reduce water and chemical usage in the removal of bifunctional reactive dyes from cotton: Part 2 bis (vinyl sulfone), aminochlorotriazine/vinyl sulfone and bis (aminochlorotriazine/vinyl sulfone) dyes. Dye. Pigment..

[24] Chakraborty J.N. (2010). Dyeing with Reactive Dye. Fundamentals and Practices in Colouration of Textiles.

[25] Chakraborty J.N. (2014). Introduction to Colouration of Textiles. Fundamentals and Practices in Colouration of Textiles.

[26] Burkinshaw S.M., Paraskevas M. (2011). The dyeing of silk: Part 3 the application and wash-off of modified vinyl sulfone dyes. Dyes Pigment..

[27] Biswas S., Huang X., Badger W.R., Nantz M.H. (2010). Nucleophilic cationization reagents. Tetrahedron Lett..

[28] Venudharan V., Chandrappa A.K., Biligiri K.P., Kaloush K.E. (2016). Predictive models for storage modulus and loss modulus of asphalt mixtures. J. Mater. Civ. Eng..

[29] Bhudolia S.K., Perrotey P., Joshi S.C. (2017). Enhanced vibration damping and dynamic mechanical characteristics of composites with novel pseudo-thermoset matrix system. Compos. Struct..

[30] Sever K., Tavman I.H., Seki Y., Turgut A., Omastova M., Ozdemir I. (2013). Electrical and mechanical properties of expanded graphite/high density polyethylene nanocomposites. Compos. Part. B Eng..

